# Quantifying Transmission of *Clostridium difficile* within and outside Healthcare Settings

**DOI:** 10.3201/eid2204.150455

**Published:** 2016-04

**Authors:** David P. Durham, Margaret A. Olsen, Erik R. Dubberke, Alison P. Galvani, Jeffrey P. Townsend

**Affiliations:** Yale School of Public Health, New Haven, Connecticut, USA (D.P. Durham, A.P. Galvani, J.P. Townsend);; Washington University School of Medicine, St. Louis, Missouri, USA (M.A. Olsen, E.R. Dubberke)

**Keywords:** Clostridium difficile, bacteria, theoretical models, transmission, asymptomatic infections, community-acquired infections, healthcare settings, nosocomial infections, hospital infections

## Abstract

To quantify the effect of hospital and community-based transmission and control measures on *Clostridium difficile* infection (CDI), we constructed a transmission model within and between hospital, community, and long-term care-facility settings. By parameterizing the model from national databases and calibrating it to *C. difficile* prevalence and CDI incidence, we found that hospitalized patients with CDI transmit *C. difficile* at a rate 15 (95% CI 7.2–32) times that of asymptomatic patients. Long-term care facility residents transmit at a rate of 27% (95% CI 13%–51%) that of hospitalized patients, and persons in the community at a rate of 0.1% (95% CI 0.062%–0.2%) that of hospitalized patients. Despite lower transmission rates for asymptomatic carriers and community sources, these transmission routes have a substantial effect on hospital-onset CDI because of the larger reservoir of hospitalized carriers and persons in the community. Asymptomatic carriers and community sources should be accounted for when designing and evaluating control interventions.

## Abstract 2

Empirical quantification of transmission dynamics for all settings is needed when evaluating interventions and control strategies.

Infection with the nosocomial pathogen *Clostridium difficile* is a major risk in healthcare settings and long-term care facilities (LTCFs) and has an increasing prevalence in the broader community. Infection is diagnosed in >250,000 hospitalized persons annually in the United States (*1*). Colonization of the gut microbiota with *C. difficile* can be innocuous and asymptomatic. However, antimicrobial drugs disrupt the normal intestinal microbial architecture and can enable proliferation of *C. difficile* (*2*). An insufficient host antibody response to *C. difficile* toxins A and B can then lead to *C. difficile* infection (CDI). CDI is a severe diarrheal disease that is concentrated among elderly persons and those with extended hospital stays or residing in LTCFs. The relative risk for CDI, given recent antimicrobial drug exposure, differs greatly among antimicrobial drug classes and ranges from no relative risk when receiving tetracyclines to a 20-fold relative risk when receiving clindamycin (*2*). Despite an increasing interest in *C. difficile* biology and the epidemiology of CDI, fundamental questions about reservoirs and routes of transmission remain unanswered.

Molecular typing and contact tracing studies have estimated that 10%–38% of CDI cases that occur >48 hours after hospital admission (termed hospital-onset CDI) can be attributed to transmission from known symptomatic contacts within the hospital (*3*–*6*). These estimates suggest that a substantial proportion of CDI arises from other sources, such as transmission from patients with asymptomatic colonization or community acquisition (*3*,*5*,*7*,*8*). The relative role of these routes of transmission to the epidemiology of *C. difficile* is crucial for determining effectiveness of hospital-based measures to control infection. In addition, toxin-targeting treatments, such as vaccines, nontoxigenic *C. difficile*, and monoclonal antibodies, might protect against CDI but are unlikely to prevent asymptomatic colonization with *C. difficile* (*9*). To predict the effectiveness of these emerging therapies, it is critical to understand the role of asymptomatic carriers in CDI epidemiology.

Mathematical models of *C. difficile* colonization have generated insights regarding the epidemiologic role of antimicrobial drugs on CDI outbreaks (*10*). Such models have also quantified the effect of hospital-based control interventions (*11*–*14*) and demonstrated the crucial roles of asymptomatic colonization and patients with exposure before hospital admission in sustaining hospital transmission (*7*,*13*). Most studies have focused on the hospital setting. To fully understand the epidemiology of the pathogen and to inform decisions regarding control strategies, it is crucial to quantify the relative transmission of *C. difficile* in the hospital and in the broader community (*8*).

To evaluate the relative role of asymptomatic hospital transmission, symptomatic hospital transmission, LTCF transmission, and community transmission, we integrated diverse clinical and epidemiologic data into a dynamic model of *C. difficile* transmission within and among hospitals, LTCFs, and community settings in the United States. We parameterized our model by using Medicare and Healthcare Cost and Utilization Project databases and data from published epidemiologic and clinical research. To estimate infectivity of symptomatic and asymptomatic patients in the hospital; corresponding infectivity of persons in LTCFs and in the community; and average risks for acquiring *C. difficile* in the hospital, LTCF, and the community, we fit our model to estimated toxigenic *C. difficile* colonization and CDI incidence in each of these settings. Furthermore, we calculated the effect on CDI incidence of targeting key aspects of CDI epidemiology with control interventions in each of the 3 settings.

## Methods

### Definitions

We refer to acquisition of *C. difficile* from human sources as *C. difficile* transmission and acquisition of *C. difficile* from nonhuman sources as nonhuman acquisition. Asymptomatic persons carrying *C. difficile* are referred to as colonized. Persons carrying *C. difficile* and symptomatic for diarrheal disease associated with *C. difficile* are referred to as persons with CDI.

### Model Structure

Previous models have focused almost exclusively on the hospital setting (*7*,*8*,*10*,*12*). We constructed a new model that encompasses *C. difficile* transmission and symptomatic CDI within a hospital, an LTCF, and an associated mid-sized community and quantifies patient movement between these settings. We parameterized our model with data from a combination of sources, including published literature, the US Census, national hospital and LTCF surveys, and the Healthcare Cost and Utilization Project and Medicare databases (Technical Appendix).

**Figure 1:**
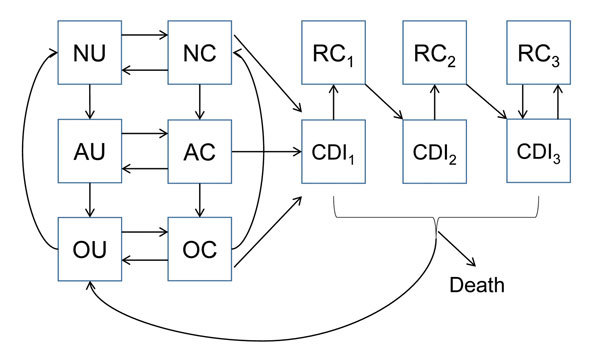
Compartmental model structure for *Clostridium difficile* infection (CDI) within each setting (hospital, long-term care facility, and community). Patients are classified as not receiving antimicrobial drugs (N), are receiving antimicrobial drugs (A), having a recent history of receiving antimicrobial drugs (O), uncolonized (U), asymptomatically colonized (C), symptomatically infected (CDI), or colonized and subject to recurrence (RC) of CDI. Arrows indicate changes in individual epidemiologic status. Subscripts indicate primary, secondary, or tertiary CDI.

We structured our model in compartments (Figure 1) composed of patients who are currently receiving antimicrobial drugs, those who have a history of antimicrobial drug use and an increased risk for CDI, or those who do not have a recent history of receiving antimicrobial drugs. Consistent with clinical observations (*15*), we assumed that the increased risk for CDI after antimicrobial drug use reverted to normal in an average of 45 days. Uncolonized patients could become asymptomatically colonized with *C. difficile* because of transmission from asymptomatic patients, transmission from patients with CDI, or through acquisition from background sources in the community. Asymptomatically colonized patients could remain asymptomatic, spontaneously clear their colonization, or develop symptomatic CDI. Patients with CDI could recover and be at temporarily increased risk for recolonization, could recover and remain colonized and at risk for recurrence, or could die from the disease. We included 3 CDI and recurrence classes, each with a successively higher likelihood of recurrence, to reflect clinical observations of the increasing likelihood of recurrence after multiple CDI episodes (*16*–*18*). We assumed that all patients with CDI were first asymptomatically colonized before symptoms developed.

**Figure 2:**
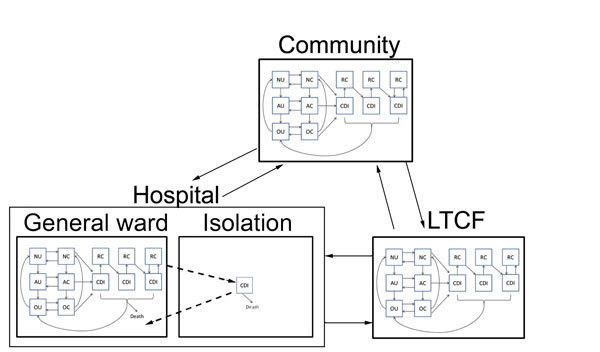
Transitions between settings (hospital, LTCF, and the non–healthcare community) for model structure of *Clostridium difficile* infection (CDI). Transitions were parameterized at demographically calibrated, age-specific rates. Hospitalized patients with CDI who were given a diagnosis are subject to enhanced isolation protocols that reduce transmission. All hospitalized CDI patients are discharged at a slower rate than non–CDI patients, which reflects longer hospitalization attributable to CDI. N, patients not receiving antimicrobial drugs; A, patients receiving antimicrobial drugs; O, patients with a recent history of receiving antimicrobial drugs; U, uncolonized patients; C, asymptomatically colonized patients; RC, symptomatically infected patients or colonized patients and subject to recurrence; LTCF, long-term care facility. Solid arrows indicate changes in individual epidemiologic status and patient movement between the hospital, community, and LTCF. Dashed arrows indicate isolation of CDI patients.

We embedded this epidemiologic model within a model of patient flow between the hospital, LTCF, and community (Figure 2), parameterized from national hospital and long-term-care-facility survey data. Patients with CDI remained hospitalized for an additional 3.1 days (95% CI 2.3–4.0 days) (*19*–*21*). Patients with CDI had a 96% (95% CI 93%–99%) probability of being given a diagnosis and subjected to isolation protocols that reduced transmission by 53% (95% CI 37%–72%) (*22*–*25*). We further assumed that persons in the community and in an LTCF in whom CDI developed were hospitalized with probabilities of 26% (95% CI 23%–28%) and 27% (95% CI 23%–32%), respectively (Table 1) (*26*,*27*).

**Table 1:** Epidemiologic and clinical model parameters for infection with Clostridium difficile*

Parameter description	Prior rate (95% CI)†	Posterior rate (95% CI)†	Reference
Epidemiology			
All-cause CDI mortality rate, %			(*28*)
Age, y			
<50	4.7 (2.6–7.6)	4.5 (2.6–7.5)	
50–64	12 (8.7–16)	12 (8.5–16)	
>65	16.6 (14–19)	17 (14–19)	
Rate at which patients complete antimicrobial drug course	0.22 (0.17–2.29)	0.22 (0.17–2.29)	(*29*)
Rate at which recurrence develops in recovered patients	0.13 (0.24–1)	0.2 (0.32–1.05)	(*30*)
Rate at which patients not receiving antimicrobial drugs at increased risk for CDI revert to normal risk	0.038 (0.012–0.062)	0.033 (0.014–0.056)	(*15*)
Rate of recovery from CDI	0.099 (0.090–0.11)	0.099 (0.092–0.11)	(*22*)
Probability that a patient recovering from primary CDI will have >1 recurrence	22 (13–34)	24 (15–36)	(*16**,**17*)
Probability that a patient recovering from a first recurrence will have a second recurrence	33 (19–48)	34 (20–48)	(*16**,**17*)
Probability that a patient recovering from multiple recurrences will have an additional recurrence	56 (42–70)	56 (41–68)	(*17**,**18*)
Relative risk for CDI developing while a patient receives antimicrobial drugs	8.9 (4.9–13.)	8.3 (4.2–12)	(*2**,**15*)
Relative risk for CDI among persons 50–65 y of age vs. those <50 y of age	2.2 (1.4–3.4)	2.2 (1.5–3.0)	(*31*)
Relative risk for CDI among persons >65 y of age compared with those <50 y of age	2.9 (1.9–4.4)	3.2 (2.1–4.3)	(*31*)
Spontaneous clearance of asymptomatic *C. difficile* colonization	0.020 (0.015–0.025)	0.021 (0.016–0.026)	(*32*)
Hospital protocols			
All-cause fraction of community-onset CDI in patients who are hospitalized	0.26 (0.23–0.28)	0.26 (0.23–0.28)	(*26*)
All-cause fraction of LTCF-onset CDI in patients who are hospitalized	0.27 (0.23–0.32)	0.27 (0.23–0.32)	(*27*)
Increased attributable length of stay for hospitalized patients with CDI	3.1 (2.3–4.0)	3.1 (2.3–4.1)	(*19**–**21*)
Effectiveness of enhanced infection control measures in reducing transmission	53 (37–72)	52 (37–68)	(*22**,**23*)
Probability that a patient with CDI is properly identified and given enhanced infection control measures	0.96 (0.93–0.99)‡	0.96 (0.94–0.99)	(*24**,**25*)
Antimicrobial drug use rates			
Prescription rate among persons in community			(*33**,**34*)
Age, y			
<50	0.0013 (0.00095–0.0017)	0.0014 (0.00095–0.0018)	
50–64	0.0014 (0.00097–0.0018)	0.0014 (0.00097–0.0017)	
>65	0.0017 (0.0013–0.0021)	0.0017 (0.0013–0.0022)	
Prescription rate among patients in hospital	0.37 (0.22–0.66)	0.37 (0.21–0.68)	(*29*)
Prescription rate among patients in LTCF	0.0054 (0.0027–0.009)	0.0052 (0.0026–0.0087)	(*35*)

*CDI, *C. difficile infection*; LTCF, long-term care facility.  †Parameter rates are per day unless otherwise indicated. ‡A total of 73% of sites initiated protocols before laboratory confirmation and 27% initiated protocols after confirmation. Sensitivity was 86% for laboratory tests, which yielded an effective diagnosis rate of 0.73 + 0.27 × 0.86 = 0.96.

### Demographics

To represent demographically stratified CDI risk between the 3 settings, we modeled 5 demographic groups: persons <50 years of age, those 50–65 years of age without concurrent conditions, those 50–65 years of age with concurrent conditions, those >65 years of age without concurrent conditions, and those >65 years of age with concurrent conditions. Therefore, our full model consisted of base epidemiology (Figure 1) applied to each of the 5 demographic groups, and each group populated and moved between the hospital, LTCF, and the community (Figure 2) at rates calibrated from published *C. difficile* literature, US hospital discharge and census data, and Medicare and Healthcare Cost and Utilization Project databases (Technical Appendix Table 4). We assumed that colonized patients with concurrent conditions are at greater risk for development of CDI (online Technical Appendix).

### Transmission

We specified 5 *C. difficile* transmission rates: 1) the base CDI rate at which patients without a diagnosis and symptomatic CDI transmit in the hospital, 2) the base asymptomatic rate at which asymptomatically colonized patients transmit in the hospital, 3) the LTCF transmission rate representing the relative infectivity of persons in LTCFs compared with patients in the hospital, 4) the community transmission rate representing the relative infectivity of persons in the community compared with patients in the hospital, and 5) the rate of *C. difficile* acquisition from nonhuman reservoirs. We further defined the force of colonization as the rate at which uncolonized patients become asymptomatically colonized with *C. difficile* and specified 3 separate force-of-colonization rates: 1) the hospital, 2) LTCF, and 3) the community.

For the force of colonization in the hospital, we specified that nonisolated symptomatic patients with CDI transmit at the base CDI rate, that isolated patients with CDI transmit at the base CDI rate multiplied by the probability that isolation measures are insufficient, and that asymptomatically colonized patients transmit at the base asymptomatic rate. We assumed direct contact mixing and density-dependent transmission, which is consistent with the observation that larger hospitals have greater CDI incidence than smaller hospitals (*36*). Environmental contamination and transmission mediated by healthcare workers were implicitly included by our calibration of the base CDI rate and the base asymptomatic rate. Hospital hygiene was separated into 2 components: overall hospital hygiene, which influenced transmission from asymptomatically colonized patients and from undiagnosed patients with CDI; and the probability of, and effectiveness of, enhanced isolation protocols for patients given a diagnosis of CDI.

For the force of colonization in the LTCF, we made 3 assumptions. First, enhanced isolation protocols were not available. Second, patients with CDI transmit at the base CDI rate multiplied by the LTCF transmission rate modifier. Third, asymptomatically colonized patients transmit at the base asymptomatic rate multiplied by the LTCF transmission rate modifier.

For the force of colonization in the community, we assumed that *C. difficile* could be acquired from nonhuman reservoirs (*37*), that patients with CDI transmit at the base CDI rate multiplied by the community transmission rate modifier, and that asymptomatically colonized patients transmit at the base asymptomatic rate multiplied by the community transmission rate modifier. Because there are insufficient published data with which to statistically differentiate between human transmission in the community and nonhuman acquisition, we estimated the force of colonization directly during our model calibration and then calculated the upper bounds for the community transmission rate modifier and for the rate of nonhuman acquisition.

Although age, history of antimicrobial drug use, and concurrent conditions are predictors of diarrheal CDI, they are not predictors of asymptomatic *C. difficile* colonization (*38**,**39*). Therefore, we assumed that the rate at which symptomatic CDI developed in colonized patients was dependent on age, antimicrobial drug use, concurrent conditions, and hospitalization status. Transmission parameters and force of colonization were independent of age, antimicrobial drug use or concurrent conditions (online Technical Appendix).

### Calibration

We used the Markov Chain Monte Carlo Metropolis algorithm (*40*) to calibrate our stochastic model and combined prior parameter densities (Table 1) with epidemiologic data, including asymptomatic prevalence and CDI incidence in the hospital, LTCF, and community Technical Appendix Table 2). This analysis yielded an ensemble of 1,000 parameter sets that estimated the joint posterior distribution for parameters with prior literature estimates (Table 1) for the 5 transmission parameters and for the base rate at which CDI developed in asymptomatically colonized persons (Table 2). Details of coding, the stochastic model, and calibration are provided in the online Technical Appendix.

**Table 2:** Calibrated posterior estimates of previously unknown epidemiologic parameters for infection with Clostridium difficile*

Parameter description	Posterior rate (95% CI)
Hospital force of colonization†	0.023 (0.017–0.032)
Base CDI transmission rate within hospital†	1.2 × 10^−2^ (0.65–2.1 × 10^−2^)
Base CDI transmission rate within hospital accounting for isolation/control measures†	6.0 × 10^−3^ (3.6–9.7 × 10^−3^)
Base asymptomatic transmission rate within hospital†	4.0 × 10^−4^ (2.4–5.5 × 10^−4^)
Relative transmission from patients with CDI compared with asymptomatically colonized patients, accounting for isolation/control measures‡	15 (7.2–32)
LTCF force of colonization†	3.7 × 10^−3^ (0.96–7.7 × 10^−3^)
LTCF transmission rate, relative to hospital‡	0.13 (0.068–0.22)
LTCF transmission rate, relative to hospital, accounting for hospital CDI isolation/control measures‡	0.27 (0.13–0.51)
Community force of colonization†	1.2 × 10^−3^ (0.50–2.3 × 10^−3^)
Community transmission rate, relative to hospital‡§	5.2 × 10^−4^ (3.3–8.9 × 10^−4^)
Community transmission rate, relative to hospital, accounting for hospital CDI isolation/control measures‡ §	1.0 × 10^−3^ (0.62–2.0 × 10^−3^)
Rate of community acquisition from nonhuman reservoirs§	1.2 × 10^−3^ (0.50–2.3 × 10^−3^)
Base rate of CDI developing in hospital†¶	2.1 × 10^−4^ (1.0–4.7 × 10^−4^)
Base rate of CDI developing in LTCF†¶	8.6 × 10^−5^ (1.1–22 × 10^−5^)
Base rate of CDI developing in community†¶	6.3 × 10^−6^ (2.9–12 × 10^−6^)
Base rate of CDI developing given concurrent conditions†¶	2.6 (0.78–6.8)

*CDI, *C. difficile* infection; LTCF, long-term care facility. †Parameter rates are per day.  ‡Parameter rate expresses relative risk.  §Parameter rate represents an upper bound on the risk for transmission or acquisition within the community. ¶For a detailed decomposition of the rate of development of CDI, see the Technical Appendix.

### Epidemiologic Analysis

To estimate relative infectivity of a hospitalized patient with CDI compared with a hospitalized asymptomatically colonized patient, accounting for isolation protocols, we computed the ratio of 1) the base CDI transmission rate from a hospitalized patient with CDI multiplied by the probability that the patient is either not given a diagnosis or that isolation protocols are improperly implemented to 2) the base asymptomatic transmission rate from a hospitalized, asymptomatically colonized patient. To generate a posterior distribution for this ratio, we repeated this calculation for each of the 1,000 runs in our posterior sample. To estimate the average risk for a person to become exposed to and colonized with *C. difficile*, for each of the runs, we computed the average force of colonization within the hospital, community, and LTCF.

To estimate an upper bound for the community transmission rate and for nonhuman acquisition, we first computed the daily average community force of colonization, which represents the sum of *C. difficile* transmission from other persons in the community plus acquisition from nonhuman reservoirs. By setting the nonhuman acquisition rate to 0, we calculated an upper bound for the community transmission rate. Likewise, by setting the community transmission rate to 0, we calculated an upper bound for nonhuman acquisition. We repeated this step for each of the 1,000 runs and generated posterior distributions for the upper bounds of the community transmission rate and the nonhuman acquisition rate.

### Control Strategy Analysis

To quantify the effect of transmission control interventions on CDI incidence, we varied each of the following factors: CDI diagnosis rate of a hospitalized patient with CDI, effectiveness of isolation protocols for a patient given a diagnosis, overall hospital hygiene, improvements in community transmission, and improvements in LTCF transmission across a range from 0 to double the model-fitted maximum likelihood estimate and while sampling all other model parameters from their posterior distributions. We used linear regression to determine the reduction for hospital-onset CDI, community-onset CDI, and LTCF-onset CDI incidence per 1% improvement in each transmission control intervention.

To compute the effect of different classes of antimicrobial drugs on CDI incidence, we varied the antimicrobial drug risk ratio in the hospital from 1, which is representative of low-risk antimicrobial drugs (e.g., tetracyclines), to 20, which is representative of high-risk antimicrobial drugs (e.g., clindamycin) (*2*). While varying the antimicrobial drug risk ratio, we sampled all other parameters, including community and LTCF antimicrobial drug risk, from their posterior distributions, thereby obtaining 95% CIs for our estimates of the effect of antimicrobial drug class on CDI incidence. We repeated this analysis for antimicrobial drug risk in the community and the LTCF. We then calculated changes in hospital-onset CDI, community-onset CDI, and LTCF CDI incidence as hospital, community, and LTCF risk for antimicrobial drug use were varied.

## Results

### Epidemiology

For within the hospital, we computed that the ratio of transmission from an isolated symptomatic patient with CDI with transmission from an asymptomatic patient was 15 (95% CI 7.2–32) (Table 2). This high ratio indicates that a symptomatic patient with CDI contributes more to transmission than does an asymptomatically colonized patient, even after accounting for *C. difficile* protocols. Within the LTCF, the transmission rate from a person with CDI to an uncolonized person is 27% (95% CI 13%–51%) that of the hospital, and the transmission rate from an asymptomatically colonized person to an uncolonized person is 13% (95% CI 6.8%–22%) that of the hospital. Within the community, the transmission rate from a person with CDI to an uncolonized person is 0.1% (95% CI 0.062%–0.2%) that of the hospital, and the transmission rate from an asymptomatically colonized person to an uncolonized person is 0.052% (95% CI 0.033%–0.089%) that of the hospital (Table 2).

To estimate the average risk for a person to become exposed to and be colonized with *C. difficile*, we computed the force of colonization. We calculated that an uncolonized person in the hospital has a probability of 2.3% (95% CI 1.7%–3.2%) per day of acquiring *C. difficile* and becoming a carrier (with or without symptoms); an uncolonized person in the community has a probability of 0.12% (95% CI 0.050%–0.23%) per day, and a person in an LTCF has a probability of 0.37% (95% CI 0.096%–0.77%) per day (Table 2). These results provide a quantitative estimate of the average risk for *C. difficile* exposure to persons in each setting.

### Control Strategy

**Figure 3:**
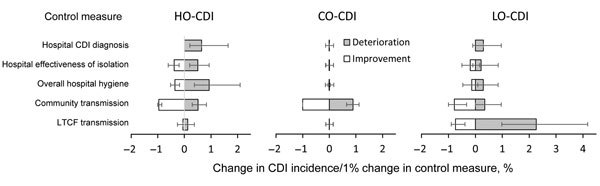
Effectiveness of *Clostridium difficile* infection (CDI) control parameters on incidence of infection quantified as percentage change in hospital-onset CDI (HO-CDI), community-onset CDI (CO-CDI), and long-term care facility (LTCF)–onset CDI (LO-CDI), quantified as percentage change in incidence per 1% change in each of 5 transmission parameters. Error bars indicate 95% CIs. LTCF, long-term care facility.

To estimate the effect of transmission control interventions on CDI incidence, we computed the percentage reduction in hospital-onset CDI, community-onset CDI, and LTCF CDI per percentage improvement in hospital CDI diagnosis rate, effectiveness of isolation protocols, overall hospital hygiene, transmission in the community, and transmission in an LTCF (Figure 3). We found that CDI diagnosis rate, effectiveness of isolation, overall hospital hygiene, and transmission in the community, but not transmission in an LTCF, affected hospital-onset CDI. In addition, community-onset CDI and LTCF CDI were not affected by hospital-based transmission interventions.

**Figure 4:**
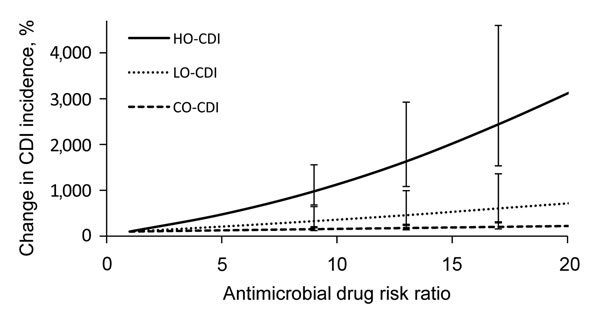
Increase in *Clostridium difficile* infection (CDI) incidence from use of antimicrobial drugs for in hospital-onset (HO-CDI), community-onset (CO-CDI), and long-term care facility–onset (LO-CDI) illnesses classified by drug risk ratio for CDI. *Clostridium difficile* infection (CDI) incidence from use of antimicrobial drugs for low through high CDI risk. Change in CDI incidence is measured as a multiple of the CDI incidence for an antimicrobial drug risk ratio = 1.0. Error bars indicate 95% CIs.

As the relative risk for antimicrobial drug class prescribed within each of the settings was increased, the CDI incidence likewise increased within that setting (Figure 4). However, there was no relationship between the antimicrobial drug class prescribed within a location and CDI incidence in another location. Specifically, we estimated that for every unit increase in antimicrobial drug risk ratio, the CDI incidence increased by 160% (95% CI 98%–320%) in the hospital, 33% (95% CI 13%–83%) in the LTCF, and 6.4% (95% CI 3.9%–13%) in the community. These results indicate that the effect of antimicrobial drug risk on CDI incidence is intertwined with *C. difficile* transmission dynamics, which differ between the hospital, LTCF, and community.

## Discussion

Through stochastic simulation and Bayesian model calibration, we estimated *C. difficile* transmission rates within and outside the healthcare setting. We also quantified the effect on CDI incidence of control interventions that reduce these transmission rates. We found that a person with CDI in an LTCF transmits at a rate 27% that for a comparable patient in the hospital, and a colonized person or a person with CDI in the community transmits *C. difficile* to others at a rate <0.1% that of a comparable patient in the hospital. Despite the lower community transmission rate, we found that because of the much larger pool of colonized persons in the community, interventions that reduce community transmission hold substantial potential to reduce hospital-onset CDI by reducing the number of patients entering the hospital with asymptomatic colonization. Moreover, our results show that in the hospital, symptomatic CDI patients under isolation and infection control measures nonetheless transmit CDI to uncolonized patients at a rate that is 15 times greater than that of asymptomatic carriers. This higher rate of transmission indicates that toxin-targeting treatments (such as vaccines); nontoxigenic *C. difficile*; and monoclonal antibodies, which might protect against symptomatic CDI but not against asymptomatic colonization, could be effective tools for reducing not only primary CDI cases but also for further transmission (*9*).

Our epidemiologic results underscore the need for incorporating and understanding transmission dynamics within and outside healthcare settings when evaluating *C. difficile* control strategies. Although *C. difficile* transmission rates are lower among asymptomatically colonized persons, residents of LTCFs, and persons in the community than in hospitalized patients with symptomatic CDI, overall CDI incidence is driven by several factors: transmission, antimicrobial drug use, and underlying population health. We found that, per unit increase in relative antimicrobial drug risk, CDI incidence increases by a factor of 160% in the hospital and 33% in the LTCF but only by a factor of 6.4% in the community. This finding is a consequence of amplification by concentration. 

When we compared patients in the hospital and LTCF with persons in the community, we found that patients are closer to each other, are more frequently receiving antimicrobial drugs, and tend to have poorer overall health or may be immunocompromised. These attributes combine to yield a greater risk for infection and transmission. This finding of amplification-by-concentration has major implications for antimicrobial drug risk management: those antimicrobial drugs strongly associated with CDI, such as clindamycin, cephalosporins, and fluoroquinolones (*2*), will have a more detrimental effect on overall CDI incidence in a high-transmission setting, such as a hospital, than they will in a moderate-transmission setting, such as an LTCF, or in a low-transmission setting, such as the community.

We found no major effect of hospital-based transmission interventions on LTCF-onset CDI or of LTCF-based transmission interventions on hospital-onset CDI. This finding suggests that although *C. difficile* can be introduced by a patient who acquired the bacteria in the hospital, CDI outbreaks in LTCFs are driven primarily from within and are best mitigated by targeted transmission interventions within the facility. Likewise, any interventions to reduce transmission within an LTCF will have limited effect on hospital-onset CDI because LTCF transmission interventions will not influence continued introduction of *C. difficile* to the hospital from the community.

The control strategies we evaluated (Figure 3) are representative of a broad range of interventions. For example, an improvement in hospital isolation effectiveness could be achieved through enhanced hospital staff adherence to precautions, or alternatively through an increased capacity to keep a patient with CDI in isolation for the duration of the disease. An improvement in the LTCF transmission rate could be achieved through an improvement to LTCF staff hygiene and cleanliness, through an increased availability of private facilities for residents, or through the isolation of LTCF residents with CDI.

Although there are few data with which to differentiate the sources of community-associated *C. difficile*, we were able to use a community *C. difficile* colonization study (*37*) to calibrate our model. From our calibrated model, we estimated the overall community force of colonization and calculated an upper bound for the community transmission rate. Future studies of similar design but with greater statistical power than the study used for our calibration (*37*), which survey healthy, nonhospitalized adults for asymptomatic *C. difficile* carriage while differentiating community risk factors, would provide the necessary data with which our model could directly quantify transmission from human sources and acquisition from nonhuman reservoirs.

Our analyses demonstrated that *C. difficile* transmission among healthcare settings and the community is interconnected, and there are comparable effects of community-based transmission and hospital-based transmission on hospital-onset CDI. We found that the effect of antimicrobial drug use on CDI incidence is modulated by transmission dynamics, with specific antimicrobial drugs exacerbating incidence, and doing so to a greater degree in high-transmission settings than in low-transmission settings. These results underscore the need for empirical quantification of community-associated transmission and the need of understanding transmission dynamics in all settings when evaluating *C. difficile* interventions and control strategies.

**Technical Appendix.** Additional information on modeling of *Clostridium difficile* infection.
