# Supplementary material for: Quantifying Transmission of Clostridium difficile within and outside Healthcare Settings
Source: Emerg Infect Dis. 2016 Apr;22(4):608–16. doi: 10.3201/eid2204.150455 (PMC4806959; doi:10.3201/eid2204.150455)
Supplement: Supplementary file 1 — Technical Appendix. Additional information on modeling of Clostridium difficile infection. [file 15-0455-Techapp-s1.pdf]

Article DOI: http://dx.doi.org/10.3201/eid2204.150455

Quantifying Transmission of *Clostridium difficile* within and outside Healthcare Settings

Technical Appendix

Supplemental Methods

Model Parameterization

Our model (Technical Appendix Figure 1) is parameterized according to the specified rates (Technical Appendix Table 1). Parameter values and CIs are provided in the main text (Table 1). *Clostridium difficile* transmission is separately modeled as the force of colonization within the hospital, within the long-term care facility (LTCF), and within the community (Equation 1). The hospital force-of-colonization was λ_H, where *g* indicates the overall hospital hygiene control parameter, CDI_H indicates the number of hospitalized patients with symptomatic *C. difficile* infection (CDI) (CDI_1 + CDI_2 + CDI_3), and C_H indicates the number of asymptomatically colonized patients in the hospital (NC + AC + OC + RC; N, patients not receiving antimicrobial drugs; C, asymptomatically colonized patients; A, patients receiving antimicrobial drugs; O, patients with a recent history of receiving antimicrobial drugs; RC, symptomatically infected patients or colonized patients and subject to recurrence). The LTCF force-of-colonization was λ_L with CDI_L and C_L representing the number of symptomatic CDI and asymptomatic colonized patients, respectively, in the LTCF. The community force-of-colonization was λ_C with CDI_C and C_C representing the number of symptomatic CDI and asymptomatic colonized patients, respectively, in the community.

Equation 1

$$\lambda_H = g\{(1-\pi)\beta_S CDI_H + \beta_A C_H\} + \pi(1-\epsilon)\beta_S CDI_H$$

$$\lambda_L = \beta_L(\beta_S CDI_L + \beta_A C_L)$$

$$\lambda_C = \beta_C(\beta_S CDI_C + \beta_A C_C) + \chi$$

Model Implementation

We used the Gibson-Bruck (*1*) adaptation of the Gillespie algorithm to run simulations coded in C++ over 2-year time horizons. To ensure a well-mixed model, we discarded the first year of results and stored results from the second year. We averaged these results for every analysis and parameter set over 10 independent runs of the model. We found 2-year time horizons with a 1-year burn-in and ten-run averages sufficient to average out the stochastic variance of the Gillespie algorithm, generating summary statistics without undue computational burden.

Model Outcome Tracking

In our stochastic model, we distinguished between *C. difficile* that was acquired in the hospital, in the community, or in the LTCF, as well as whether that acquisition was caused by transmission from a person with a CDI, from an asymptomatic carrier, or from nonhuman acquisition. By storing this information, we identified for every new CDI case where that case originated. We then computed the proportion of hospital-onset CDI that was caused by transmission from other patients with CDI (Technical Appendix Table 2).

Model Initial Conditions

We initialized our model with an endemic *C. difficile* colonization prevalence in the hospital, LTCF, and community (Technical Appendix Table 2). We specified a total population of 100,000 persons distributed according to age, concurrent condition, and location (Technical Appendix Table 3).

Model Calibration

To estimate unknown parameters, we fit our model to a range of epidemiologic and demographic data. We divided model parameters and epidemiologic outcomes into 3 categories: 1) those for which extensive data are available, which we used to fit the model; 2) those for which extensive data are available, which we used to validate the fitted model; and 3) those for which little data are available, which we estimated from the fitted model.

We specified data-driven prior values and 95% CIs for each parameter for which data are available (Table 1 in main text), as well as for epidemiologic outcomes, such as CDI incidence and asymptomatic colonization (Technical Appendix Table 2). We fit our model by using a Markov Chain Monte Carlo (MCMC) simulation. The MCMC simulation proceeded by

generating a candidate estimate for each unknown parameter (Table 2 in main text), simultaneously sampled from prior distributions of the known parameters (Table 1 in main text), and then ran the model under the candidate parameter set. The candidate parameter set, which included samples from known and unknown parameters, was accepted or rejected according to the Metropolis algorithm (*12*). We based the Metropolis objective function upon the log-likelihood of the epidemiologic outcomes, which was defined as the sum of the logs of the target distributions for the epidemiologic outcomes (Technical Appendix Table 2), and evaluated at the candidate parameter set. Using a computing cluster, we generated 100 independent MCMC chains of 10,000 runs each. We discarded the first 2,000 runs of each chain, visually confirmed convergence of the likelihood and of each model parameter, and thinned each chain at equally spaced intervals to obtain a final ensemble of 1,000 runs.

Model Validation

Our model predicted that 0.16% (95% CI 0.10%–0.23%) of hospital admissions had symptomatic CDI, which was consistent with current estimates for the United States (*13,14*). We calculated the source of acquisition for hospital-onset CDI cases, and separated these cases into 3 groups. Our calibrated model predicted that 29% (95% CI 19%–41%) of cases were acquired from another symptomatic CDI patient in the hospital, 49% (95% CI 32%–62%) were acquired from an asymptomatically colonized patient in the hospital, and 22% (95% CI 12%–35%) were among patients who entered the hospital with endogenous *C. difficile* colonization and in whom diarrheal CDI subsequently developed during their hospital stay. These results are consistent with findings from molecular typing and contact tracing, which estimate 30%–35% of hospital-onset from symptomatic patients and at least 45% of hospital-onset CDI from asymptomatic contacts or from nonhospital-transmission sources (*11,15*).

Model Demographics

We parameterized population distribution, non-CDI deaths, and patient movement from published *C. difficile* literature, the Healthcare Cost and Utilization Project Nationwide Inpatient Sample, the Centers for Medicare and Medicaid 5% random sample Chronic Conditions Warehouse database (Medicare), the Truven Health Analytics MarketScan (MarketScan), the Healthcare Cost and Utilization Project State Inpatient Databases, US hospital discharge and long-term-care survey reports, and US Census data. We specified a total population size of

100,000 persons. We used the Elixhauser definition for concurrent conditions (*16,17*) and excluded hypertension because of its high prevalence among elderly persons (*18*).

Population Distribution

To quantify the percentage of the population that is hospitalized and in each of the age stratifications (Technical Appendix Table 3), we estimated from the National Hospital Discharge Survey that 37.4%, 19.5%, and 43.0% of hospital patient-days are occupied by those <50, 50–65, and >65 years of age, respectively (*19*). We anchored this age breakdown to our estimate that, at any given time, 1.06% of the US population >65 years of age is hospitalized (Medicare). We combined these estimates with our estimates that, in the hospital, 55% of patients 50–65 years of age and 79% of patients >65 years of age have concurrent conditions (Medicare, MarketScan).

To quantify the percentage of the US population that is in an LTCF, we estimated that 1.38 million persons in the United States reside in LTCFs at any point in time, of whom 85.1% are >65 years of age (*20*). We assumed that the remaining 14.9% are 50–65 years of age. We estimated that 90.14% of LTCF residents have concurrent conditions (Medicare).

To estimate the population breakdown of the United States that lives in the community (e.g., not hospitalized or in an LTCF), we calculated the population remaining according to our hospital and LTCF calculations and stratified this community population according to US Census age profiles and our estimate that 54.97% of persons >65 years of age and 23.74% of persons 50–65 years of age the general community have concurrent conditions (Medicare, Marketscan, 67).

Patient Movement

To estimate rates of movement between the hospital, LTCF, and community (Technical Appendix Table 4), we calculated the hospital discharge rate, LTCF discharge rate, and LTCF discharge destination from published sources (*19,21*). We estimated the fraction of hospital discharges that are sent to an LTCF vs. those sent to home (Medicare, Healthcare Cost and Utilization Project State Inpatient Databases). We assumed that 40.5% of LTCF residents were admitted from a hospital, and we used this value to calculate the rate of admission from the community (*22*). Finally, we calculated the hospitalization and LTCF admission rates from the community that would produce an equilibrium population distribution. Because of limited data, we parameterized LTCF discharge rates independently of age or concurrent conditions.

Non-CDI Death Rate

From the National Hospital Discharge Survey, we estimated a death rate of 0.0016 deaths/day among inpatients <50 years of age, 0.0034 deaths/day among inpatients 50–65 years of age, and 0.0073 deaths/day among inpatients >65 years of age. From the US Census and the National Hospital Discharge Survey, we estimated that among the 210 million persons <50 years of age, there are 252,000 annual deaths, of which 97,000 occur within a hospital (*19*,*23*) (Technical Appendix Table 5). For the 65 million persons 50–65 years of age, there are 506,000 annual deaths, of which 109,000 occur within a hospital. For the 40 million persons >65 years of age, there are 1.8 million annual deaths, of which 525,000 occur within a hospital. We assumed that for persons <50 and 50–65 years of age, all nonhospital deaths occur in the community, which yields a daily mortality rate of 2.0×10^{-6} and 1.7×10^{-5}, respectively. For persons >65 years of age, we estimated that 39% of all deaths occur in home or hospice care (*24*), which yields a daily mortality rate in the community of 5.1×10^{-5}. We estimated a daily LTCF mortality rate among persons >65 years of age of 0.0020.

Parameter Assumptions and Derivation

Rate (μ) at Which Symptomatic CDI Develops in Asymptomatically Colonized Patients

We partitioned μ into components by age, concurrent condition, antimicrobial drug history, and hospitalization status. First, we specified that asymptomatically colonized persons <50 years of age without concurrent conditions and with no recent antimicrobial drug use showed development of CDI at a base rate of μ_C, μ_H, or μ_L, which reflected current residence and underlying health in the community, in the hospital, or in the LTCF, respectively. When we controlled for all other factors, we found that colonized patients 50–65 years of age were parameterized to be μ_{50} times as likely to show development of CDI as those <50 years of age. Colonized patients >65 years of age were parameterized to be μ_{65} times as likely to show development of CDI as those <50 years of age (*25*). Second, we parameterized colonized persons with current or recent antimicrobial drug use history (AC or OC) to be μ_A times as likely to show development of CDI as those without such exposure (*26*–*30*). Finally, persons with concurrent conditions were parameterized to be μ_m times as likely to show development of CDI as those without concurrent conditions. Thus, for a colonized patient 50–65 years of age with concurrent

conditions, currently in the hospital and taking antimicrobial drugs, the daily risk for CDI developing would be $\mu_H\mu_{50}\mu_m\mu_A$.

Hospital-Onset CDI Calculation

We calculated the number of patients with hospital-onset CDI as the sum of the number of patients with *C. difficile* acquired in the hospital with symptom onset in the hospital, plus the number of patients with *C. difficile* acquired outside the hospital with symptom onset in the hospital >48 hours after admission. To estimate the probability that a patient colonized at hospital admission shows development of symptoms while in the hospital, and does so $\geq$48 hours after hospital admission, we solved the subset of model equations given below, with boundary conditions NC(0) = 1, AC(0) = OC(0) = CDI(0) = 0. Thus, CDI(*t*) gives the probability that a patient entering the hospital, with *C. difficile* colonization acquired outside the hospital and without recent antimicrobial drug use (NC), will show development of CDI while in the hospital (Technical Appendix Figure 2).

$$\frac{\mathbf{dNC}(t)}{\mathbf{d}t} = -(\phi + \mu + m_{\mathrm{H}} + \delta + d_{\mathrm{H}})\mathbf{NC}(t)$$

$$\frac{\mathbf{dAC}(t)}{\mathbf{d}t} = \phi\mathbf{NC}(t) - (\rho + \mu\mu_{\mathrm{A}} + m_{\mathrm{H}} + \delta + d_{\mathrm{H}})\mathbf{AC}(t)$$

$$\frac{\mathbf{dOC}(t)}{\mathbf{d}t} = \rho\mathbf{AC}(t) - (\mu\mu_{\mathrm{A}} + m_{\mathrm{H}} + \delta + d_{\mathrm{H}})\mathbf{OC}(t)$$

$$\frac{\mathbf{dCDI}(t)}{\mathbf{d}t} = \mu\mu_{\mathrm{A}}\mathbf{AC}(t) + \mu\mu_{\mathrm{A}}\mathbf{OC}(t) + \mu\mathbf{NC}(t)$$

The closed form solution of CDI(*t*), the probability that an NC (colonized at admission, but not taking antimicrobial drugs) patient will show development of CDI in the hospital by day *t*, is given by

$$\mathbf{CDI}(t) = \frac{-\left(\mu\left(e^{-t(o+\mu+\phi)}(-1+\mu_{\mathrm{A}})(o+\mu\mu_{\mathrm{A}})(\mu+\phi) - e^{-t(o+\mu\mu_{\mathrm{A}})}\mu_{\mathrm{A}}\phi(o+\mu+\phi) - (\mu(-1+\mu_{\mathrm{A}})-\phi)(o+\mu_{\mathrm{A}}(\mu+\phi))\right)\right)}{\left((o+\mu\mu_{\mathrm{A}})(\mu(-1+\mu_{\mathrm{A}})-\phi)(o+\mu+\phi)\right)}$$

We define the outflow parameter $o = m_{\mathrm{H}} + \delta + d_{\mathrm{H}}$ to simplify the notation. CDI(∞) provides the probability that an NC patient will show development of CDI during the hospital stay, and 1 – CDI(∞) provides the probability that an NC patient will spontaneously clear colonization, die, or be discharged before development of CDI. Because CDI(2) gives the

probability that an NC patient will show development of CDI during the first 2 days of hospitalization, it follows that CDI(∞) – CDI(2) gives the probability of development of CDI ≥2 days after admission. We compute the probability P_2 that a patient, colonized at hospital admission and with CDI onset in the hospital, will show development of symptoms ≥2 days after hospital admission. We then use P_2 to compute the total rate of hospital-onset.

$$P_2 = P(CDI\ onset \geq two\ days\ after\ admission \mid CDI\ onset\ during\ hospital\ stay) = \frac{\mathrm{CDI}(\infty) - \mathrm{CDI}(2)}{\mathrm{CDI}(\infty)} = 1 - \frac{\mathrm{CDI}(2)}{\mathrm{CDI}(\infty)}$$
$$= \frac{(e^{-2(o+\mu+\phi)}(1-\mu_A)(o+\mu\mu_A)(\mu+\phi) + e^{-2(o+\mu\mu_A)}\mu_A\phi(o+\mu+\phi)}{(\mu(1-\mu_A)+\phi)(o+\mu_A(\mu+\phi))}$$

References

1. Gibson MA, Bruck J. Efficient exact stochastic simulation of chemical systems with many species and many channels. J Phys Chem A. 2000;104:1876–89. http://dx.doi.org/10.1021/jp993732q

2. Loo VG, Bourgault A-M, Poirier L, Lamothe F, Michaud S, Turgeon N, et al. Host and pathogen factors for *Clostridium difficile* infection and colonization. N Engl J Med. 2011;365:1693–703. PubMed http://dx.doi.org/10.1056/NEJMoa1012413

3. Guerrero DM, Becker JC, Eckstein EC, Kundrapu S, Deshpande A, Sethi AK, et al. Asymptomatic carriage of toxigenic *Clostridium difficile* by hospitalized patients. J Hosp Infect. 2013;85:155–8. PubMed http://dx.doi.org/10.1016/j.jhin.2013.07.002

4. Kyne L, Warny M, Qamar A, Kelly CP. Asymptomatic carriage of *Clostridium difficile* and serum levels of IgG antibody against toxin A. N Engl J Med. 2000;342:390–7. PubMed http://dx.doi.org/10.1056/NEJM200002103420604

5. Ziakas PD, Zacharioudakis IM, Zervou FN, Grigoras C, Pliakos EE, Mylonakis E. Asymptomatic carriers of toxigenic *C. difficile* in long-term care facilities: a meta-analysis of prevalence and risk factors. PLoS ONE. 2015;10:e0117195. PubMed http://dx.doi.org/10.1371/journal.pone.0117195

6. Galdys AL, Nelson JS, Shutt KA, Schlackman JL, Pakstis DL, Pasculle AW, et al. Prevalence and duration of asymptomatic *Clostridium difficile* carriage among healthy subjects in Pittsburgh, Pennsylvania. J Clin Microbiol. 2014;52:2406–9. PubMed http://dx.doi.org/10.1128/JCM.00222-14

7. Lessa FC, Mu Y, Winston L, Dumyati G, Farley MM, Beldavs Z, et al. Determinants of *Clostridium difficile* infection across diverse U.S. geographic locations. Open Forum Infect Dis. 2014;1:ofu048.

8. Dubberke ER, Butler AM, Yokoe DS, Mayer J, Hota B, Mangino JE, et al. Multicenter study of *Clostridium difficile* infection rates from 2000 to 2006. Infect Control Hosp Epidemiol. 2010;31:1030–7. PubMed http://dx.doi.org/10.1086/656245

9. Campbell RJ, Giljahn L, Machesky K, Cibulskas-White K, Lane LM, Porter K, et al. *Clostridium difficile* infection in Ohio hospitals and nursing homes during 2006. Infect Control Hosp Epidemiol. 2009;30:526–33. PubMed http://dx.doi.org/10.1086/597507

10. Pawar D, Tsay R, Nelson DS, Elumalai MK, Lessa FC, Clifford McDonald L, et al. Burden of *Clostridium difficile* infection in long-term care facilities in Monroe County, New York. Infect Control Hosp Epidemiol. 2012;33:1107–12. PubMed http://dx.doi.org/10.1086/668031

11. Curry SR, Muto CA, Schlackman JL, Pasculle AW, Shutt KA, Marsh JW, et al. Use of multilocus variable number of tandem repeats analysis genotyping to determine the role of asymptomatic carriers in *Clostridium difficile* transmission. Clin Infect Dis. 2013;57:1094–102. PubMed http://dx.doi.org/10.1093/cid/cit475

12. Metropolis N, Rosenbluth AW, Rosenbluth MN, Teller AH, Teller E. Equation of state calculations by fast computing machines. J Chem Phys. 1953;21:1087. http://dx.doi.org/10.1063/1.1699114

13. Zilberberg MD. Increase in adult *Clostridium difficile*–related hospitalizations and case-fatality rate, United States, 2000–2005. Emerg Infect Dis. 2008;14:929–31. PubMed

14. McDonald LC, Owings M, Jernigan D. *Clostridium difficile* infection in patients discharged from US short-stay hospitals, 1996–2003. Emerg Infect Dis. 2006;12:409–15. PubMed

15. Eyre DW, Cule ML, Wilson DJ, Griffiths D, Vaughan A, O'Connor L, et al. Diverse sources of *C. difficile* infection identified on whole-genome sequencing. N Engl J Med. 2013;369:1195–205. PubMed http://dx.doi.org/10.1056/NEJMoa1216064

16. Elixhauser A, Steiner C, Harris DR, Coffey RM. Comorbidity measures for use with administrative data. Med Care. 1998;36:8–27. PubMed http://dx.doi.org/10.1097/00005650-199801000-00004

17. Comorbidity software. Healthcare cost and utilization project, 2014 [cited 2015 Dec 29]. http://www.hcup-us.ahrq.gov/toolssoftware/comorbidity/comorbidity.jsp

18. Kearney PM, Whelton M, Reynolds K, Muntner P, Whelton PK, He J. Global burden of hypertension: analysis of worldwide data. Lancet. 2005;365:217–23. PubMed http://dx.doi.org/10.1016/S0140-6736(05)70151-3

19. CDC/NCHS. National Hospital Discharge Survey, 2010 [cited 2015 Dec 29]. http://www.cdc.gov/nchs/nhds.htm

20. Harris-Kojetin L, Sengupta M, Park-Lee E, Valverde R. Long-term care services in the United States: 2013 overview. Vital Health Stat 3. 2013; (37):1–107. PubMed

21. Arling G, Kane RL, Cooke V, Lewis T. Targeting residents for transitions from nursing home to community. Health Serv Res. 2010;45:691–711. PubMed http://dx.doi.org/10.1111/j.1475-6773.2010.01105.x

22. Jones AL, Dwyer LL, Bercovitz AR, Strahan GW. The National Nursing Home Survey: 2004 overview. Vital Health Stat 13. 2009;(167):1–155. PubMed

23. US Census Bureau. National Population Projections, 2014 [cited 2015 Dec 29]. https://www.census.gov/population/projections/data/national/2014.html

24. Teno JM, Gozalo PL, Bynum JPW, Leland NE, Miller SC, Morden NE, et al. Change in end-of-life care for Medicare beneficiaries: site of death, place of care, and health care transitions in 2000, 2005, and 2009. JAMA. 2013;309:470–7. PubMed http://dx.doi.org/10.1001/jama.2012.207624

25. Dubberke ER, Reske KA, Olsen MA, McMullen KM, Mayfield JL, McDonald LC, et al. Evaluation of *Clostridium difficile*–associated disease pressure as a risk factor for *C difficile*-associated disease. Arch Intern Med. 2007;167:1092–7. PubMed http://dx.doi.org/10.1001/archinte.167.10.1092

26. Dial S, Kezouh A, Dascal A, Barkun A, Suissa S. Patterns of antibiotic use and risk of hospital admission because of *Clostridium difficile* infection. CMAJ. 2008;179:767–72. PubMed http://dx.doi.org/10.1503/cmaj.071812

27. Hensgens MP, Goorhuis A, Dekkers OM, Kuijper EJ. Time interval of increased risk for *Clostridium difficile* infection after exposure to antibiotics. J Antimicrob Chemother. 2012;67:742–8. PubMed http://dx.doi.org/10.1093/jac/dkr508

28. Marwick CA, Yu N, Lockhart MC, McGuigan CC, Wiuff C, Davey PG, et al. Community-associated *Clostridium difficile* infection among older people in Tayside, Scotland, is associated with antibiotic exposure and care home residence: cohort study with nested case-control. J Antimicrob Chemother. 2013;68:2927–33. PubMed http://dx.doi.org/10.1093/jac/dkt257

29. Kuntz JL, Chrischilles EA, Pendergast JF, Herwaldt LA, Polgreen PM. Incidence of and risk factors for community-associated *Clostridium difficile* infection: a nested case–control study. BMC Infect Dis. 2011;11:194. PubMed http://dx.doi.org/10.1186/1471-2334-11-194

30. Deshpande A, Pasupuleti V, Thota P, Pant C, Rolston DD, Sferra TJ, et al. Community-associated *Clostridium difficile* infection and antibiotics: a meta-analysis. J Antimicrob Chemother. 2013;68:1951–61. PubMed http://dx.doi.org/10.1093/jac/dkt129

Technical Appendix Table 1. Parameter names and symbols used for the model of *Clostridium difficile* infection*

Parameter description	Symbol
Epidemiology	
All-cause CDI mortality	α
Rate at which patients complete antimicrobial drug course	ρ
Rate at which recovered patients show recurrence	q
Rate at which patients not receiving antimicrobial drugs and at increased CDI risk revert to normal risk	θ
Rate of recovery from CDI	γ
Probability that a patient recovering from primary CDI will have at least 1 recurrence	r
Probability that a patient recovering from a first recurrence will have a second recurrence	r_2
Probability that a patient recovering from multiple recurrences will have additional recurrence	r_3
Relative risk for development of CDI while receiving antimicrobial drugs	μ_A
Relative risk for CDI among persons 50–65 years of age vs. those <50 years of age	μ_{50}
Relative risk for CDI among persons >65 years of age vs. those <50 years of age	μ_{65}
Spontaneous clearance of asymptomatic *C. difficile* colonization	δ
Hospital protocols	
All-cause fraction of community-onset CDI that are hospitalized	τ
All-cause fraction of LTCF-onset CDI that are hospitalized	τ_L
Increased attributable length of stay for hospitalized patients with CDI	d_{CDI}
Effectiveness of enhanced infection control measures in reducing transmission	ε
Probability that a patient with CDI is identified and given enhanced infection control measures	π
Antimicrobial drug rates	
Prescription rate among persons in the community	ϕ_C
Prescription rate among patients in the hospital	ϕ_H
Prescription rate among patients in the LTCF	ϕ_L
Transmission	
Hospital force of colonization	λ_H
Community force of colonization	λ_C
LTCF force of colonization	λ_L
Base CDI transmission rate within the hospital	β_S
Base asymptomatic transmission rate within the hospital	β_A
LTCF transmission rate relative to hospital	β_L
Community transmission rate relative to hospital	β_C
Rate of community acquisition from nonhuman reservoirs	χ
Overall hospital hygiene	g

*Parameter values and CIs are provided in the main text. CDI, *C. difficile* infection; LTCF, long-term care facility.

Technical Appendix Table 2. Epidemiologic data used to compose the likelihood function for the MCMC simulation of *Clostridium difficile* infection*

Clinical and epidemiologic data	Estimate (95% CI)	Likelihood distribution	Reference
Asymptomatic hospital colonization prevalence	11% (5.6%–18%)	Gamma (11.7, 106)	(*2*–*4*)
Asymptomatic colonization in LTCF	14.8% (7.6%–24%)	Normal (0.148, 0.0418)	(*5*)
Asymptomatic colonization among healthy adults in community	6.6% (2.8%–12%)	Beta (7, 99)	(*6*)
Community-onset CDI†			(*7*)
Overall	37.7 (18.6–56.8)‡	Normal (37.7, 9.72)	
Age 50-64 years	50.4 (46–55)	Normal (50.4, 2.24)	
Age ≥65 years	114.4 (104–124)	Normal (114, 5.2)	
Hospital-onset rate CDI†	7.6 (5.7–9.8)§	Gamma (52.9, 6.98)	(*8*,*9*)
Hospital recurrence	1.6 (0.24–2.9)§	Normal (1.55, 0.67)	(*8*)
LTCF-onset incidence†	2.3 (0–5.3)§	Normal (2.25, 1.56)	(*9*,*10*)
LTCF recurrence	0.85 (0–2.4)§	Normal (0.85, 0.815)	(*9*,*10*)
Proportion of hospital-onset cases attributable to other CDI patients	30% (19%–43%)	Beta (17, 39)	(*11*)

*MCMC, Markov Chain Monte Carlo; LTCF, long-term care facility; CDI, *C. difficile* infection.
†Excludes recurrent cases.
‡Units of cases/100,000 person-years.
§Units of cases/10,000 patient-days.

Technical Appendix Table 3. Normalized demographic population breakdown in the United States for *Clostridium difficile* infection

Age, y	Hospital	Community	Long-term care facility
<50	1.2×10^{-3}	0.66	0
50–65 without concurrent conditions	2.7×10^{-4}	0.16	6.5×10^{-5}
50–65 with concurrent conditions	3.4×10^{-4}	0.049	5.9×10^{-4}
>65 without concurrent conditions	2.8×10^{-4}	0.056	3.7×10^{-4}
>65 with concurrent conditions	1.1×10^{-3}	0.069	3.4×10^{-3}

Technical Appendix Table 4. Rate of patient movement between hospital, LTCF, and community, United States*

Parameter description	Symbol	Age, y, rate/day
Hospital discharge to community	d_{HC}	<50: 0.22; 50–65 without concurrent conditions: 0.18; 50–65 with concurrent conditions: 0.18; >65 without concurrent conditions: 0.16; >65 with concurrent conditions: 0.15
Hospital discharge to LTCF	d_{HL}	<50: 0; 50–65 without concurrent conditions: 0.00086; 50–65 with concurrent conditions: 0.0028; >65 without concurrent conditions: 0.0056; >65 with concurrent conditions: 0.0095
LTCF admission from community	d_{CL}	<50: 0; 50–65 without concurrent conditions: 2.2×10^{-6}; 50–65 with concurrent conditions: 2.8×10^{-5} ; >65 without concurrent conditions: 4.2×10^{-5}; >65 with concurrent conditions: 0.00021
Hospital admission from community	d_{CH}	<50: 0.00038; 50–65 without concurrent conditions: 0.00031; 50–65 with concurrent conditions: 0.0013; >65 without concurrent conditions: 0.00078; >65 with concurrent conditions: 0.0024
Discharge from LTCF to community	d_{LC}	0.0056
Discharge from LTCF to hospital	d_{LH}	0.00032

*LTCF, long-term care facility.

Technical Appendix Table 5. Age-specific mortality rates for non–*Clostridium difficile* infections for hospital, LTCF, and community, United States*

Parameter description	Symbol	Age, y, rate/day
Non-CDI mortality rate in hospital	m_H	<50: 0.0016; 50–64: 0.0033; >65: 0.0073
Non-CDI mortality rate in LTCF	m_L	<50: 0; 50–64: 0; >65: 0.0020
Non-CDI mortality rate in community	m_C	<50: 2.0×10^{-6}; 50–64: 1.7×10^{-5}; >65: 5.1×10^{-5}

*CDI, *C. difficile infection*; LTCF, long-term care facility.

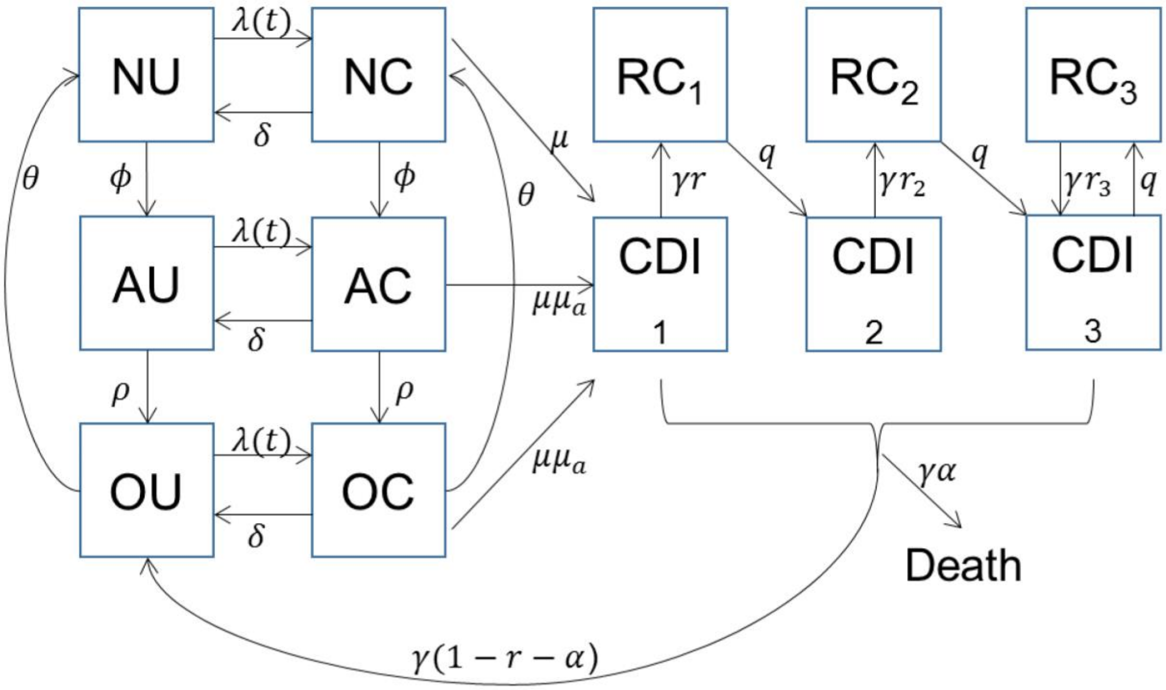

Technical Appendix Figure 1. *Clostridium difficile* infection model flowchart, including parameters. N, patients not receiving antimicrobial drugs; U, uncolonized patients; C, asymptomatically colonized patients; RC, symptomatically infected patients or colonized patients and subject to recurrence; A, patients receiving antimicrobial drugs; CDI, *C. difficile* infection; O, patients with a recent history of receiving antimicrobial drugs. Arrows indicate changes in individual epidemiologic status. Subscripts indicate primary, secondary, or tertiary CDI.

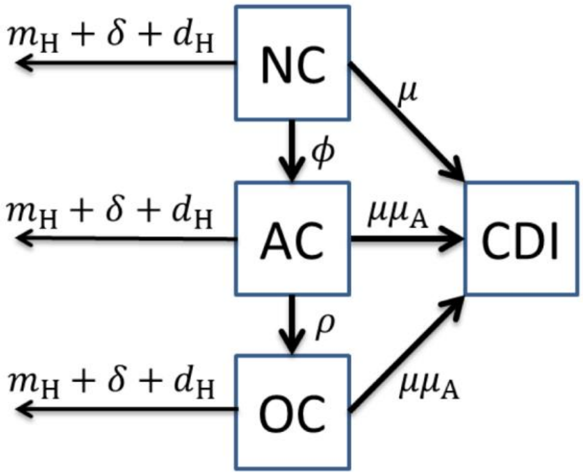

Technical Appendix Figure 2. Submodel of a larger model of *Clostridium difficile* infection (CDI), which was used separately to mathematically estimate the proportion of cases with colonization outside of the hospital but with diarrheal CDI arising in the hospital that are classified as hospital onset (occurring $\geq$48 hours after hospital admission). N, patients not receiving antimicrobial drugs; C, asymptomatically colonized patients; A, patients receiving antimicrobial drugs; O, patients with a recent history of receiving antimicrobial drugs. Arrows indicate changes in individual epidemiologic status.
